# [Retracted] CtBP1 interacts with SOX2 to promote the growth, migration and invasion of lung adenocarcinoma

**DOI:** 10.3892/or.2023.8588

**Published:** 2023-06-15

**Authors:** Yifei Liu, Tingting Bian, Jia Feng, Li Qian, Xiaoli Li, Qing Zhang, Jianguo Zhang, Daishan Jiang, Jian Liu, Jiahai Shi

Oncol Rep 42: 67–78, 2019; DOI: 10.3892/or.2019.7142

Following the publication of this paper, it was drawn to the Editors' attention by a concerned reader that, for the western blots showing the CtBP1 and SOX2 bands in Fig. 5C on p. 74, the data were in fact the same, but flipped horizontally; moreover, two pairs of overlapping data panels were identified comparing between the cell invasion and assay data images shown in Figs. 3E and 6C, such that these were likely to have been derived from the same original sources even though they were intended to show the results from differently performed experiments; similarly, the ‘shSOX2 / 24 h’ and ‘shCtBP1 / 24 h’ data panels in Fig. 6B showing the results of differently performed scratch-wound assay experiments appeared to be overlapping, albeit with one of the panels being slightly rotated relative to the other. Finally, there were erroneous calculations included for the CtBP1 expression data shown in Table III.

Given the large number of apparent errors that were made during the assembly of various of the figures and Table III in this paper, the Editor of *Oncology Reports* has decided that this paper should be retracted from the Journal due to an overall lack of confidence in the presented data. After contacting the authors, they accepted the decision to retract this paper. The Editor apologizes to the readership for any inconvenience caused.

